# Giant Omphalocele Treated With Intramuscular Tissue Expansion

**Published:** 2014-01-16

**Authors:** Jacob Buinewicz, Donald Laub

**Affiliations:** University of Vermont, Burlington, Vt

**Keywords:** abdominal wall, congenital, omphalocele, tissue expansion, Ventral Hernia

A 6-year-old girl had undergone multiple operations for an omphalocele but still retains a very large ventral hernia. She presented with an eventrated abdomen with unstable, scarred closure (see Fig. 1). A reconstruction using intermuscular tissue expanders was performed.

## QUESTIONS

1. **What defines an omphalocele as giant?**
2. **What are the different techniques employed to close giant omphaloceles?**
3. **Is there a benefit to a staged approach compared to a primary closure?**
4. **What are the risks associated with the use of tissue expanders?**

## DISCUSSION

A giant omphalocele is a congenital abdominal wall defect caused by failure of the cephalic, caudal, and lateral folds to fuse at the umbilical ring during the fourth week of gestation.^1^ Fifty percent of infants with omphalocele also have other abnormalities, either structural or chromosomal.^2^ Staged closure, external silo reduction, and subcutaneous or intermuscular tissue expanders are surgical options employed for closure. In this article, we present one case of intermuscular tissue expansion for salvage of an omphalocele cripple, with a cosmetic and functional long-term outcome. There is no concrete definition of what makes an omphalocele giant; however, a defect having a diameter larger than 4 to 6 cm with the liver in the central position is widely accepted.^2^^-^4 Other definition omphalocele is dependent on is the degree of hypoplasia of the peritoneal cavity.^5^ There is a high recurrence rate associated with surgical treatment of this condition. Compression of viscera and possible respiratory and vascular compromise are also possible complications of the treatment.^4^^,^^6^

There are numerous surgical techniques for giant omphalocele closure, which fall into the categories of primary, staged, and delayed closure.^7^ A delayed form of closure involved the use of a skin-flap with delayed hernia repair.^1^ Epithelization using silver sulfadiazine cream has also an option when the defect has been greater than 10 cm in diameter and primary closure is not possible.^2^ Toxicity issues with silver sulfadiazine cream due to the absorptiveness of the amniotic sac have come to light; however, nanocrystalline silver dressings may avoid this.^8^ Both techniques are favorable for high-risk cases, but only defer definitive closure.

Staged techniques of closure require slow reintroduction of the herniated viscera back into the abdominal cavity using: external silo reduction, sequential sac ligation, external skin closure, and vacuum-assisted closure systems. External silo reduction has been attempted with both polypropylene mesh with an intact amniotic sac.^6^^,^^9^^,^^10^ The intact amniotic sac provides protection from infection; however, correction of intestinal malrotation is not possible, and a subsequent hernia repair is needed.^9^ Sequential sac ligation, using the amniotic sac as a natural silo, has also been successfully carried out by Shinohara, but has not been widely adopted due to the assumed fragility of the sac.^11^ In addition to above established methods, both external skin closure and vacuum-assisted closure techniques have shown promise in staged closure, but the process may entail an indeterminate number of operations over an unforeseen amount of time.^12^^,^^13^

Primary closure of a giant omphalocele is executed through the 2-stage use of tissue expanders: subcutaneously, intra-abdominally, or intramuscularly. Ugarte et al^14^ advocated the placement of crescent-shaped tissue expanders positioned between the internal oblique and transversus abdominis to reclaim abdominal domain. Placement of the tissue expander in the peritoneal cavity is beneficial because it creates a flap that includes all the layers of the abdominal wall; however, overinflation of the expander could potentially lead to visceral ischemia and respiratory complications.^4^ Adetayo et al^15^ reported that placing the tissue expanders subcutaneously or intramuscularly may not be possible with severe loss of domain, and intra-abdominal placement may be the only option.

Our experience with intermuscular tissue expanders leads us to agree with the case evidence provided by De Ugarte et al and Adetayo et al that intramuscular tissue expansion is an effective, low risk method of closure in the salvage situation. Tissue expansion has temporary inconvenience to the patient, possibility of infection, and the possibility of loss of domain from postponing the repair.

Omphalocele treatment approaches vary due to the varying sizes of defects and associated anomalies. A follow-up survey of giant omphalocele repair case studies published from 1967 to 2009 determined that 42% of surgeons have changed surgical technique.^7^ For the closure giant omphalocele, the use of intermuscular tissue expanders should be considered.

## Figures and Tables

**Figure 1:**
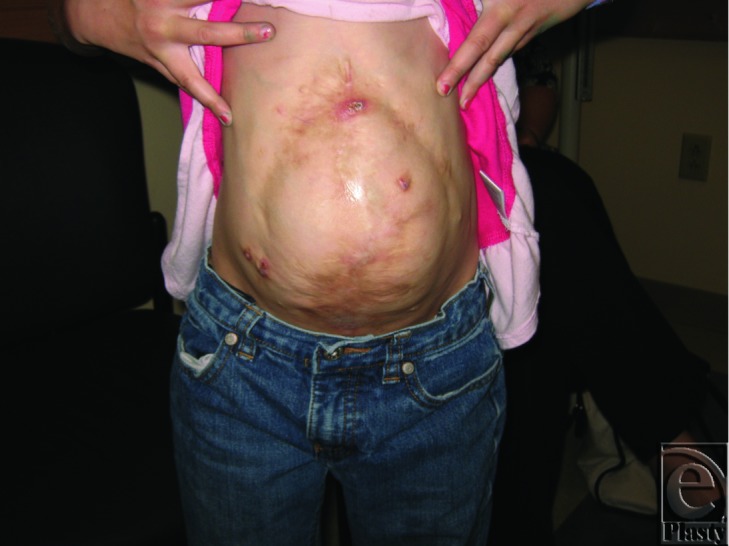
Patient presenting at age 6 after several surgical attempts at closure. She has unstable skin graft over allogenic dermis graft.

**Figure 2:**
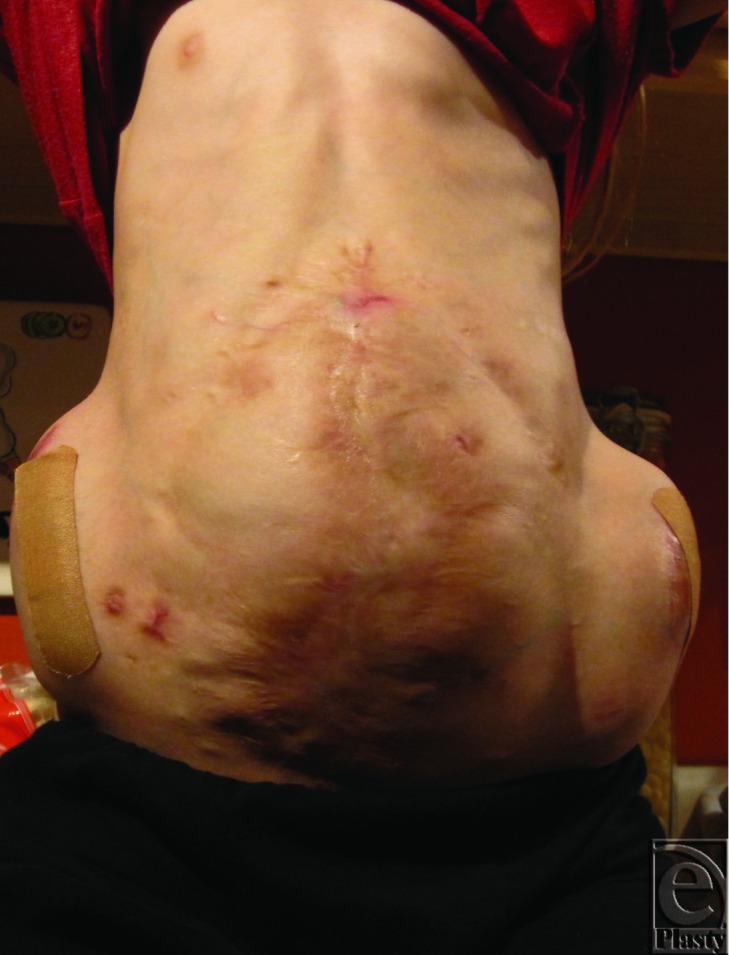
The patient with tissue expanders in place, below her external oblique muscular layer.

**Figure 3:**
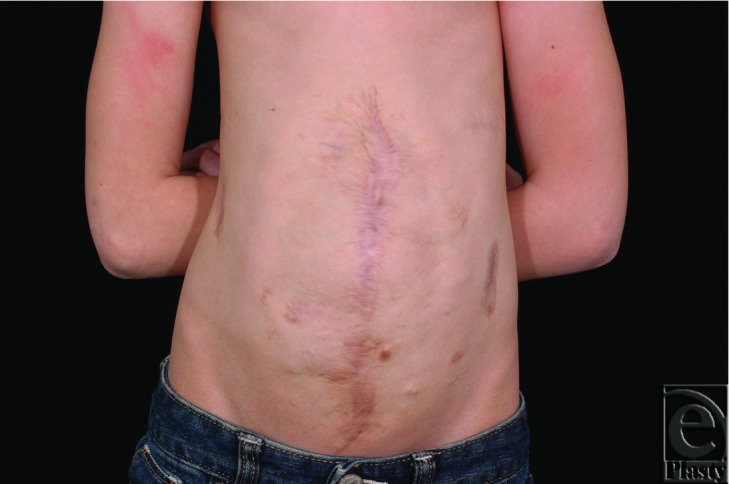
The patient at age 8, after abdominal closure. She is able to participate in dance and gymnastics classes.
